# Comparative study of MAFLD as a predictor of metabolic disease treatment for NAFLD

**DOI:** 10.1038/s41598-024-64301-3

**Published:** 2024-06-11

**Authors:** Jin Imai, Shinji Takashimizu, Nana Suzuki, Kana Ohshinden, Kana Sawamoto, Yusuke Mishima, Kota Tsuruya, Yoshitaka Arase, Mitsuhiko Yamano, Noriaki Kishimoto, Chizumi Yamada, Nagamu Inoue, Kengo Moriyama, Akiyasu Baba, Hidekazu Suzuki, Tatehiro Kagawa, Yasuhiro Nishizaki

**Affiliations:** 1 Department of Clinical Health Science, Tokai University School of Medicine, 143 Shimokasuya, Isehara, Kanagawa Japan; 2 Department of Gastroenterology, Tokai University School of Medicine, Isehara, Kanagawa Japan

**Keywords:** Metabolic associated fatty liver disease (MAFLD), Non-alcoholic fatty liver disease (NAFLD), Fatty liver, Metabolic disease, Diagnosis, Hepatology, Metabolic disorders, Non-alcoholic fatty liver disease

## Abstract

A novel concept of Metabolic Associated Fatty Liver Disease (MAFLD) was proposed, incorporating metabolic abnormalities such as obesity and diabetes, which are risk factors that affect the prognosis. Non-Alcoholic Fatty Liver Disease (NAFLD), entails fat accumulation in the liver without alcohol consumption and is often linked to obesity, insulin resistance, and metabolic syndrome. However, the broad nature of the disease concept has hindered prognosis accuracy. In this study, we assess the contribution of the impact of diagnostic criteria for MAFLD on metabolic disease progression compared to conventional diagnostic criteria for NAFLD. A total of 7159 patient who were presented to the health screening center in Tokai University Hospital both in 2015 and 2020 were included in the study. Fatty liver was diagnosed using abdominal ultrasonography. The diagnostic criteria for NAFLD were consistent with the global guidelines based on alcohol consumption. The diagnostic criteria for MAFLD were based on the International Consensus Panel. Medications (anti-hypertensive, diabetic, and dyslipidemia medications) were evaluated by self-administration in the submitted medical questionnaire. A total of 2500 (34.9%) participants were diagnosed with fatty liver (FL +), 1811 (72.4%) fit both NAFLD and MAFLD diagnostic criteria (overlap), 230 (9.2%) fit only the NAFLD diagnostic criteria (NAFLD group) and 404 (16.1%) fit the MAFLD diagnostic criteria (MAFLD group) at 2015. Over the next 5 years, medication rates increased in the NAFLD group for anti-hypertensive, + 17 (7.4%); diabetes, + 3 (1.3%); and dyslipidemia, + 32 (13.9%). In contrast, the only-MAFLD group showed a more significant increase with + 49 (12.1%), + 21 (5.2%), and + 49 (12.1%), for the respective medications, indicating a substantial rise in patients starting new medications. Our analysis of repeated health check-ups on participants revealed that the diagnostic criteria for MAFLD are more predictive of future treatment for metabolic disease than conventional diagnostic criteria for NAFLD.

## Introduction

Metabolic dysfunction-related fatty liver disease was previously defined as non-alcoholic fatty liver disease (NAFLD), excluding alcohol-related cases. It affects approximately 25% of the world’s adult population and represents a significant health and economic burden to all societies^1,2^, with the highest prevalence in South Asia, the Middle East, and South America and the lowest in Africa^2,3^. In 60% of NAFLD patients, non-alcoholic steatohepatitis (NASH) is associated with inflammatory infiltrate and progressive fibrosis^4,5^. The large patient volume with NAFLD distinguishes it from other liver diseases, emphasizing the primary focus of clinical care on identifying individuals at the highest risk of progressive liver disease^1^. Additionally, a portion of NAFLD is linked to metabolic diseases such as hypertension and diabetes mellitus, posing an elevated risk for cardiovascular and renal dysfunction and hepatic damage progression^6–9^. However, the heterogeneity of the NAFLD patient population concerning its major precipitating and co-existing disease-modifying factors represents a significant hurdle to identifying highly effective drug therapies^10^, owing that NAFLD diagnosis involves exclusionary diagnoses such as hepatitis virus and alcohol consumption. Therefore, the International Consensus Panel introduced new diagnostic criteria for metabolic (dysfunction) associated fatty liver disease (MAFLD) in 2020^10^. The criteria for MAFLD identifies metabolic deregulatory factors as a prerequisite for the diagnosis^11^. Unlike the current usage of NAFLD as a “non” disease classification, the term MAFLD, as proposed, denotes a multisystem disorder for diagnostic purposes^12^. A recent report showed that MAFLD identifies patients with significant hepatic fibrosis better than NAFLD^13^; however, it is unclear whether the criteria defined by MAFLD better capture the clinical characteristics of fatty liver patients and especially the long-term outcome of metabolic disease compared to NAFLD. Hence, this study aimed to assess the impact of MAFLD diagnostic criteria on the progression of metabolic diseases other than NAFLD, utilizing data from routine health checkups that allowed us to track the course over 5 years.

## Methods

### Participants and methods

This study is a retrospective analysis of the electronic medical records of 7159 patients, (4004 males and 3155 females), who were presented to the Health Screening Center in Tokai University Hospital, Japan both in 2015 and 2020. The fatty liver (FL) diagnosis was made through abdominal ultrasonography, employing five different parameters: liver-to-kidney contrast, liver parenchymal brightness, bright vessel walls, deep beam attenuation, and gallbladder wall definition^12^. The diagnostic criteria for NAFLD were defined by the presence of a fatty liver, diagnosed through ultrasonography in participants without an alternative cause for secondary liver steatosis, such as excessive alcohol consumption (> 30 g per day for men or > 20 g per day for women), autoimmune liver disease or positive hepatitis B and C viral infection. The diagnostic criteria for MAFLD were based on the guidelines of the International Consensus Panel^10,14^. Although, insulin resistance score and Oral glucose tolerance test (OGTT) and plasma high-sensitivity C-reactive protein levels considered in the diagnosis of MAFLD are metabolic risk abnormalities, but these data were not available in our dataset. The definition of overlap group is referenced the previous reports^12,15–17^. This group was defined as subjects with fatty liver combined with “obesity,” “type 2 diabetes,” or “two or more metabolic disorders,” and male alcohol consumption of 30 g/day or less and 20 g/day or less for women. Medications (anti-hypertensive, diabetic, and dyslipidemia medications) were evaluated through a self-administered medical questionnaire submitted by the participants.

### Statistical analysis

We used SPSS Version 19.0 (SPSS, Chicago, Illinois, USA) for statistical analysis. Continuous data was summarized using means and standard deviations. Categorical data was presented in relative frequencies and counts, and the categorical outcomes were analyzed using the χ^2^ test. The comparison of normally distributed continuous variables was analyzed using the Student’s t-test analysis. Statistical significance was set as P ≤ 0.05.

### Ethics approval and consent to participate

This study was performed in accordance with the World Medical Association Declaration of Helsinki. Participants were recruited on an opt-out basis. All data, including medical history, physical measurements, and blood tests were collected anonymously from the medical records of Tokai University Hospital. The privacy of participants was completely protected by unlikable anonymization. This study was approved by the Ethics Committee of Tokai University (17R-221). Informed consent is waived in view that this is a retrospective study by the Ethics Committee of Tokai University.

## Results

### The population of MAFLD, NAFLD, and overlapped both diagnoses

**Figure 1:**
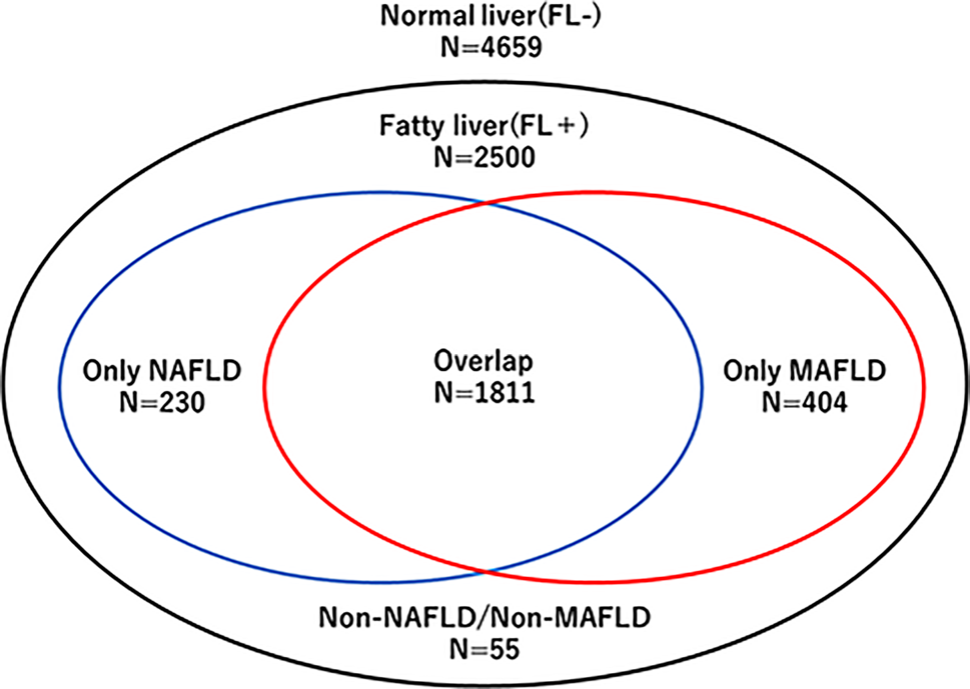
Distribution of study population in the diagnostic criteria for MAFLD and NAFLD. Fatty liver was present in 2500 of all patients while 4659 patients had no fatty liver. There were 1811 patients in the Overlap group who met both diagnostic criteria for NAFLD and MAFLD. There were 230 patients in the group that met only the diagnostic criteria for NAFLD (NAFLD/non-MAFLD) and 404 patients in the group that met only the diagnostic criteria for MAFLD (MAFLD/non-NAFLD).

The participant characteristics are summarized in Fig. 1. Out of all patients, 2500 had fatty liver, while 65% (4659) did not have fatty liver. There were 1811 patients in the overlap group who met both diagnostic criteria for NAFLD and MAFLD. Moreover, there were 230 patients in the group that met only the diagnostic criteria for NAFLD (NAFLD/non-MAFLD) and 404 patients in the group that met only the diagnostic criteria for MAFLD (MAFLD/non-NAFLD). Then, we compared the MAFLD/non-NAFLD group, which was not covered by conventional diagnostic criteria to the NAFLD/non-MAFLD group. In this study, patients were asked to describe their alcohol consumption using a self-administered method. Unfortunately, 55 patients did not complete the drinking history and therefore were not included in the diagnostic criteria (non-NAFLD/non-MAFLD group).

### Clinical and biochemical characteristics of patients with NAFLD/non-MAFLD group and MAFLD/non-NAFLD group

Participants with MAFLD/non-NAFLD exhibited higher body weight, BMI, and waist circumference, along with elevated serum liver enzymes such as aspartate aminotransferase (AST), alanine aminotransferase (ALT), γ-glutamyl transpeptidase (γ GTP) and lactate dehydrogenase (LDH) and an unfavorable metabolic profile such as fasting glucose, HbA1c, triglyceride (TG), and low HDL, compared to their NAFLD/non-MAFLD counterparts (Table 1). However, there was no significant difference in creatinine levels between the two groups. The medical history data from 2015 indicated that participants with MAFLD/non-NAFLD had significantly higher frequencies of hypertension, diabetes, and dyslipidemia compared to their NAFLD/non-MAFLD counterparts (Table 2). The result of MAFLD/non-NAFLD was at a higher level compared to total fatty liver (FL +) and also to the overlap group.

**Table 1:** Characteristics of NAFLD/non-MAFLD vs MAFLD/non-NAFLD.

	NAFLD/non-MAFLD (N = 230)				MAFLD/non-NAFLD (N = 404)				*P* value
	Mean	± SD	Min	Max	Mean	± SD	Min	Max	
Age (y)	55.8	9.9	34	82	55.9	9.1	35	82	0.053
Height (cm)	166.7	8.1	147.5	187.1	167.7	7.9	146.7	191.7	0.592
Weight (kg)	60	6.9	43.8	77.7	72.8	11.5	39.8	120.2	< 0.001
BMI	21.5	1.2	18	22.9	25.8	3.2	17.7	39.6	< 0.001
West circumstances (cm)	78.8	4.4	68.2	89.1	88.6	7.6	68.1	120.9	< 0.001
s BP (mmHg)	116.4	16.5	85	185	127.7	16.9	77	183.1	0.278
d BP (mmHg)	76.6	11.9	51	124	84.8	12.4	43	132	0.379
WBC(/μL)	5533.1	1492.5	2800	11,300	5779.9	1451.2	2400	12,000	0.654
Hb(g/dL)	14.5	1.3	8.5	16.9	14.9	1.3	10.4	18.5	0.61
PLT (/μL)	24.5	5.8	14.6	48.9	23.6	5.5	11.8	64.9	0.258
Total bilirubin (mg/dL)	0.9	0.4	0.3	3.4	0.9	0.4	0.3	2.8	0.306
GOT (U/L)	21.1	5.6	11	61	26.9	12.8	9	132	< 0.001
GPT (U/L)	21.1	8.2	6	58	30.5	17.2	8	132	< 0.001
γ-GTP (U/L)	36.6	44.4	8	556	69	72.2	12	566	< 0.001
LDH (U/L)	175	25.9	110	269	183.9	30.3	118	290	0.023
ALP (U/L)	212.1	51.6	104	418	201.8	57.9	89	557	0.22
Total protein (g/dL)	7.38	0.4	6.1	9	7.47	0.4	6.5	8.7	0.695
Albumin (g/dL)	4.3	0.4	3.5	5	4.3	0.3	3.5	5.2	0.403
Fasting glucose (mg/dL)	97.5	8.4	58	127	108.5	23.7	78	298	< 0.001
HbA1c (%)	5.4	0.25	4.9	6.4	5.7	0.7	4.6	11.5	< 0.001
Total collesterol (mg/dL)	212	32.8	99	318	205.8	32.7	117	356	0.86
HDL (mg/dL)	63.2	14.3	37	123	59.3	17.3	30	139	0.037
LDL (mg/dL)	137.1	30.7	26	227	127.1	29.4	30	216	0.579
Triglycerides (mg/dL)	102.1	48.9	25	353	153.7	99.8	37	1040	< 0.001
CRP (mg/dL)	0.1	0.2	0	2	0.2	0.2	0	3.9	0.001
Ureic acid (mg/dL)	5.7	1.2	2.8	9.5	6.2	1.3	0.9	10.8	0.053
BUN (mg/dL)	13.4	3.1	8	26	13.4	3.2	6	28	0.402
Creatine (mg/dL)	0.85	0.14	0.41	1.29	0.85	0.155	0.42	1.41	0.409

**Table 2:** Difference between total NAFLD and total MAFLD in the number of treatment patients in 2015.

	Total NAFLD (N = 2041)	Total MAFLD (N = 2215)	*P* value	Fatty liver (-) (N = 4658)
Number of people taking antihypertensive medications	726 (35.5%)	875 (39.5%)	0.01	909(19.5%)
Number of people taking diabetes medications	188 (9.2%)	225 (10.1%)	0.32	141(3.0%)
Number of people taking hyperlipidemic medications	610 (29.8%)	704 (31.8%)	0.19	773(16.6%)

### Prospective difference in the number of treatment patients for metabolic disease over five years in 2020 (Δnumber of patients)

Next, we assessed the progression of fatty liver longitudinally. First, we analyzed metabolic risk factors for the transition from normal to fatty liver over a 5 year period. The patients who progress to fatty liver already have a high BMI and tend to have high blood pressure, blood glucose, hyperlipidemia and uric acid, although within the normal range (Table 3).

**Table 3:** Metabolic risk factors for transitioning from the normal liver to fatty liver during 5 years.

	FL(-) → FL(-)(N = 3912)		FL(-) → FL( +)(N = 747)		*P* value
	Mean	± SD	Mean	± SD	
Age (y)	57.3	11.3	57.2	10.5	0.81
Height (cm)	161.9	8.7	164.3	9	< 0.001
Weight (kg)	56.2	9.4	62.5	9.8	< 0.001
BMI	21.3	2.4	23.1	2.5	< 0.001
West circumstances (cm)	76.2	7	81.4	6.6	< 0.001
s BP (mmHg)	117	17.3	120.1	17	< 0.001
d BP (mmHg)	75.5	11.9	78.4	11.9	< 0.001
WBC (/μL)	4951.7	1360.2	5535.6	1672.6	< 0.001
Hb (g/dL)	13.6	1.3	14.2	1.3	0.13
PLT (/μL)	22.9	5.5	23.9	5.8	0.15
Total bilirubin (mg/dL)	0.8	0.3	0.8	0.3	0.51
GOT (U/L)	21.1	6.4	21.4	13.8	0.25
GPT (U/L)	17.7	12.2	20.6	20.3	< 0.001
γ-GTP (U/L)	27.4	34.1	33.9	38.1	< 0.001
LDH (U/L)	181.3	31.7	181.7	38.4	0.96
ALP (U/L)	199.8	59.1	205.8	56.8	< 0.001
Total protein (g/dL)	7.3	0.4	7.3	0.3	0.695
Albumin (g/dL)	4.2	0.2	4.2	0.2	0.42
Fasting glucose (mg/dL)	96.4	13.1	99.2	12.8	< 0.001
HbA1c (%)	5.4	0.3	5.5	0.3	0.11
Total collesterol (mg/dL)	203.4	30.9	205.5	32.1	0.21
HDL (mg/dL)	71.8	16.5	63.5	15.3	< 0.001
LDL (mg/dL)	122.4	27.7	128.2	30.2	< 0.001
Triglycerides (mg/dL)	82.8	45.6	108.5	66.9	< 0.001
CRP (mg/dL)	0.1	0.3	0.1	0.2	0.82
Ureic acid (mg/dL)	5	1.2	5.7	1.2	< 0.001
BUN (mg/dL)	13.8	3.8	13.6	3.3	0.22
Creatine (mg/dL)	0.7	0.3	0.8	0.1	0.1

In Table 2, the diagnostic criteria for MAFLD indicated a significantly higher current history of metabolic disease. Therefore, we hypothesized that this data would be progressive. To address this question, we compared the rate of increase in medications over the next 5 years. The results showed that the NAFLD/non-MAFLD group exhibited increases of + 17 (7.4%) and + 3 (1.3%) for anti-hypertensive and diabetes medications, respectively. In contrast, the MAFLD/non-NAFLD group showed substantial increases of + 49 (12.1%) and + 21 (5.2%) for anti-hypertensive and diabetes medications indicating a marked increase in the number of patients taking new medications (Table 4). However, the number of patients receiving dyslipidemia medications showed no significant differentiation between the two groups: + 32 (13.9%) in NAFLD groups and + 49 (12.1%) in MAFLD groups. As considering the possibility that new medications may not reflect all metabolic disorders, we tried to analyzed the deferential cases that meet the criteria for hypertension, hyperlipidemia, and diabetes, even though new medications. Referring to the same diagnostic criteria, hypertension was defined as over 130/85 mmHg, diabetes mellitus was defined as HbA1c of 6.5% or higher, and dyslipidemia as HDL of 40 mg/dl or lower for men and 50 mg/dl or lower for women^15^. The results of this analysis showed the same results as in Table 3, hypertension and diabetes mellitus were a trend toward an increase in those meeting the diagnostic criteria for MAFLD (Supply Table 1). Moreover, Possible predictors of metabolic disease between total MAFLD and total NAFLD were analyzed (Table 5). Interestingly, there was no difference in the increase in new medicated patients among them. In other words, the effect of patients in the overlapping groups is quite large. In summary, these results suggest that meeting the diagnostic criteria for MAFLD may be more likely to progress to hypertension and diabetes mellitus.

**Table 4:** Prospective difference in the number of treatment patients in 2020 (Δnumber of patients).

	NAFLD/non-MAFLD (N = 230)	MAFLD/non-NAFLD (N = 404)	*P* value	Fatty liver (-)(N = 4658)	Fatty liver( +)(N = 2500)	Overlap(N = 1811)
Number of people taking antihypertensive medications	17 (7.4%)	49 (12.1%)	0.04	335 (7.2%)	284 (11.4%)	214 (11.8%)
Number of people taking diabetes medications	3(1.3%)	21(5.2%)	0.01	52(1.1%)	109(4.3%)	85(4.7%)
Number of people taking hyperlipidemic medications	32 (13.9%)	49 (12.1%)	0.3	387 (8.3%)	319 (12.7%)	238 (13.1%)

**Table 5:** Prospective difference between total NAFLD and total MAFLD in the number of treatment patients in 2020 (Δnumber of patients).

	total NAFLD (N = 2041)	total MAFLD (N = 2215)	*P* value
Number of people taking antihypertensive medications	231 (11.3%)	263 (11.9%)	0.71
Number of people taking diabetes medications	88 (4.3%)	106 (4.8%)	0.5
Number of people taking hyperlipidemic medications	270 (13.2%)	287 (12.9%)	0.82

## Discussion

MAFLD is not only about fat accumulation in the liver, but also a metabolic risk factor that contributes to the development and progression of the disease, including obesity, insulin resistance, type 2 diabetes mellitus, and dyslipidemia^10^. The term MAFLD is used to facilitate an accurate diagnosis and management. Therefore, this study redefines and evaluates fatty liver patients using both new MAFLD diagnostic criteria and NAFLD criteria. This allows for a detailed evaluation of patient populations that have been overlooked by conventional diagnostic criteria for NAFLD. The results of this study show that the group that met the diagnostic criteria for MAFLD/non-NAFLD had more significant metabolic disorders compared to the NAFLD/non-MAFLD group. Additionally, the 5-year longitudinal data indicate the need for therapeutic intervention in managing these metabolic diseases, particularly hypertension and diabetes. In contrast, dyslipidemia showed no difference in the patient population that met MAFLD/non-NAFLD diagnostic criteria. Metabolic disorders pose a risk for chronic kidney disease and cardiovascular events, new fatty liver diagnosis may contribute to patient outcomes. Recent reports suggest that MAFLD identifies patients with chronic kidney disease better than NAFLD^18^, and similar findings are supported by a nationwide cohort study^19^. Furthermore, a Korean nationwide cohort study with a mean duration of 10 years indicated that MAFLD is a better identifier for patients with cardiovascular disease than NAFLD^20^. Thus, the redefinition of fatty liver appears to contribute to mortality factors beyond cirrhosis. Although this study did not precisely track the occurrence of chronic kidney disease or cerebrovascular events, our focus was on metabolic disorders. Interestingly, there was no difference in the increase in the number of new medicated patients between total NAFLD and total MAFLD. The overlap group, which is a large percentage of fatty liver, has a background of metabolic disorders not caused by alcohol consumption. Conversely, we can conclude that a group of patients who meet only the diagnostic criteria for MAFLD, that is, patients with fatty liver and metabolic disease factors and who also consume a large amount of alcohol, should be given particular caution.

There have been several reports of direct hepatic effects. The diagnostic criteria for MAFLD should be a better indicator of liver fibrosis than conventional NAFLD^15,21^. We also would have liked to observe the effect of liver fibrosis, but we did not have elastography records, so we evaluated the fib-4 index. To prevent overdiagnosis due to age, we evaluated patients under 65 years of age^22^, the diagnostic criteria for MAFLD still showed a much more progressive fib-4 index (Supply Table 2). Further, A multi-population study from Geneva, Switzerland, showed that while there is an upward trend in the number of patients with HCC caused by fatty liver, the frequency of cases meeting the diagnostic criteria for MAFLD is also high^23^. Therefore, viral hepatitis and alcoholic hepatitis can also be defined as MAFLD. Among patients with chronic hepatitis B, MAFLD was associated with liver-related events and death^24^.

This study had some limitations. First, was the inaccurate survey of medications due to the self-reporting system. However, we believe that this shortfall is compensated for by the large number of participants included in the study. Second, there was no evaluation of fibrosis of the liver, such as elastography. Liver injuries, including NASH, lead to liver fibrosis, which is a separate prognostic factor from metabolic diseases. Therefore, a parallel study assessing liver fibrosis over time would be more valuable. Therefore, further research is required to address the challenge.

Since a new name; metabolic dysfunction-associated steatotic liver disease (MASLD) instead of NAFLD and metabolic dysfunction-associated steatohepatitis (MASH) instead of NASH, were recommended in an attempt to link the names to cardiometabolic risk^25^, the relationship between metabolic diseases and fatty liver will gain increased attention. Therefore, the results of this study will be used as a basis for further research.

## Conclusion

This study demonstrated that the diagnostic criteria for MAFLD are more predictive of future treatment for metabolic disease than conventional diagnostic criteria for NAFLD. Furthermore, the relationship between metabolic diseases and fatty liver will continue to be studied along with the proposal of a new disease concept called MASLD and MASH.

### Supplementary Information

Supplementary Table 1.

Supplementary Table 2.

## Data Availability

The datasets used and/or analyzed during the current study are available from the corresponding author on reasonable request.

## Abbreviations

MAFLD: Metabolic associated fatty liver disease
NAFLD: Non-alcoholic fatty liver disease
NASH: Non-alcoholic steatohepatitis
MAFLD: Metabolic dysfunction-associated fatty liver disease
MASH: Metabolic dysfunction-associated steatohepatitis
BS: Blood sugar
TG: Triglyceride
AST: Aspartate aminotransferase
ALT: Alanine aminotransferase
γGTP: γ-Glutamyl transpeptidase
LDH: Lactate dehydrogenase
